# Role Played by Receptors for Advanced Glycosylation End Products in Corneal Endothelial Cells after HSV-1 Infection

**DOI:** 10.3390/ijms22115833

**Published:** 2021-05-29

**Authors:** Dai Miyazaki, Michiko Kandori-Inoue, Yumiko Shimizu, Fumie Ohtani, Ikuyo Chono, Yoshitsugu Inoue, Satoru Yamagami

**Affiliations:** 1 Division of Ophthalmology and Visual Science, Faculty of Medicine, Tottori University, Yonago, Tottori 683-8504, Japan; micham0522@yahoo.co.jp (M.K.-I.); nognog@kjc.biglobe.ne.jp (Y.S.); f_ohtani2013@yahoo.co.jp (F.O.); applelover28@yahoo.co.jp (I.C.); yoinoue@med.tottori-u.ac.jp (Y.I.); 2 Department of Ophthalmology, Nihon University School of Medicine, Tokyo 173-8610, Japan; yamagami.satoru@nihon-u.ac.jp

**Keywords:** receptor for advanced glycosylation end products, advanced glycation end products, corneal endothelial cell, toll-like receptor 9, herpes simplex virus

## Abstract

Senescence, sterile inflammation, and infection cause dysfunction of corneal endothelial cells, leading to visual morbidity that may require corneal transplantation. With increasing age, the extracellular matrix is modified by non-enzymatic glycation forming advanced glycation end products (AGEs). The modifications are primarily sensed by the receptors for the AGEs (RAGE) and are manifested as a type I interferon response. Interestingly, in our study, human corneal endothelial cells (HCEn) cells did not respond to the typical RAGE ligands, including the AGEs, high mobility group box 1 (HMGB1), and serum amyloid-A (SAA). Instead, HCEn cells responded exclusively to the CpG DNA, which is possessed by typical corneal pathogen, herpes simplex virus-1 (HSV-1). Upon HSV-1 infection, the surface expression of RAGE was increased, and endocytosed HSV-1 was associated with RAGE and CpG DNA receptor, TLR9. RAGE DNA transfection markedly increased interferon-β secretion by CpG DNA or HSV-1 infection. HSV-1 infection-induced interferon-β secretion was abolished by TLR9 inhibition and partially by RAGE inhibition. Global transcriptional response analysis confirmed that RAGE and TLR9 were both significantly involved in type I interferon responses. We conclude that RAGE is a sensor of HSV-1 infection and provokes a type I interferon response.

## 1. Introduction

Corneal opacity is one of the major causes of blindness in the world [1,2]. In 2005, 1.3 million people were affected by corneal blindness, which is defined by a best-corrected visual acuity (BCVA) of <20/400 [1,2]. Important causes of corneal blindness in developing countries are active keratitis and corneal scars [3]. In addition, bullous keratopathy, failed corneal grafts, and corneal dystrophy have become more prevalent as causes of corneal blindness [3].

The corneal endothelium is an essential component for maintaining corneal clarity, and it does this by governing the fluid exchange and dehydration of the cornea. A loss or damage of corneal endothelial cells leads to bullous keratopathy that then results in severe visual impairment. 

Atherosclerosis, cardiovascular diseases, age-related macular degeneration, and Alzheimer’s disease are prevalent in older individuals [4]. All of these pathologies have been shown to be associated with the formation of advanced glycation endproducts (AGEs) [5,6,7]. AGEs are formed in vivo as non-enzymatic heterogeneous compounds from the oxidization of glucose and lipids in the aging processes or in oxidative environments [5,6,7]. AGEs are resistant to proteasomal degradation and need to be removed to avoid unnecessary stress responses or tissue malfunctions [6,7].

In the eyes of elderly individuals, the AGEs are present in the cornea, especially in Descemet’s membrane and corneal endothelial cells [8], and it has been suggested that they are important factors associated with the dysfunction of corneal endothelial cells.

The receptor for the AGEs (RAGE) is a pattern-recognition receptor that belongs to the immunoglobulin superfamily. AGE-bound RAGE is endocytosed for lysosomal degradation and systemic clearance from the body. RAGE is also involved in the regulation of complex homeostatic processes, and it is not limited to a role as a scavenger receptor of age-related oxidative products. RAGE also recognizes other molecules or damage-associated molecular patterns [9]. These ligands are released or are formed during infections, inflammation, aging, and cellular stress, and they induce pro-inflammatory responses. Thus, RAGE regulates various cellular processes, including inflammation, antimicrobial responses, apoptosis, cell adhesion, stress responses, and autophagy. 

An important function of corneal endothelial cells is to maintain the clarity of the cornea. Corneal endothelial insufficiency is often caused by surgical intervention, Fuchs’ endothelial corneal dystrophy (FECD), or viral infection during senescence. This leads to significant visual morbidity and bullous keratopathy [3,8,10,11,12]. Importantly, AGE is known to accumulate in corneal endothelial cells of patients with FECD [8]. However, it has not been determined how corneal endothelial cells use RAGE to respond to various adverse conditions. 

Herpes simplex virus type1 (HSV-1) is a common pathogen that infects the cornea, and more than one-half of elderly individuals are seropositive for HSV-1 [13]. In healthy subjects, a spontaneous shedding of HSV-1 is often observed in the tears [14]. HSV-1 reactivation causes a loss in corneal clarity as well as corneal endothelial cell loss in elderly individuals. In vitro studies have shown that corneal endothelial cells are highly permissive to HSV-1 infections [15,16]. 

To understand the role played by the RAGE in corneal endothelial cells, the responsiveness of human corneal endothelial cells (HCEn) to senescence-related molecules, inflammation, and viral pathogens was examined. A viral infection or inflammation often culminates in interferon production [15,16]. 

Thus, the purpose of this study was to determine the contribution of the RAGE ligands to the type I interferon responses of corneal endothelial cells, and to analyze the role that RAGE plays in maintaining the integrity of corneal endothelial cells.

## 2. Results

**Figure 1:**
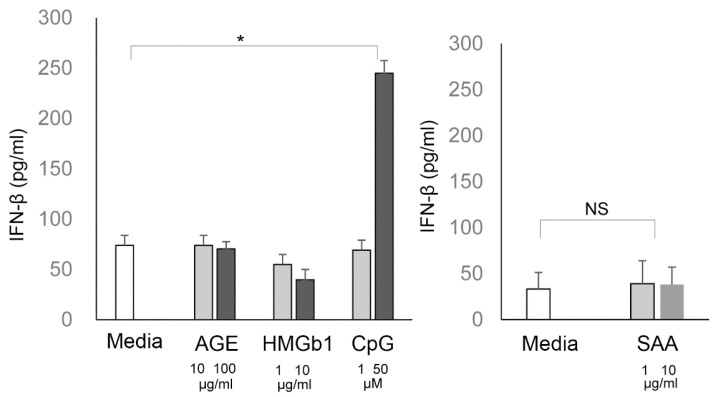
Responsiveness of human corneal endothelial (HCEn) cells to the receptors of advanced glycation end products (RAGE) ligands assessed by the level of expression of interferon-β. Exposure to CpG DNA significantly induces interferon-β expression after 12 h (left). Advanced glycation end products (AGE), HMGB1, and serum amyloid A (SAA) (right) do not significantly induce interferon-β. DMEM with 10% FBS was used for the assay media. Left and right panels were obtained from different batches of cells of single origin. For * *p* < 0.00001; *n* = 6. Ns: not significant.

To examine the roles played by the RAGE in corneal endothelial cells, we tested a panel of RAGE ligands on their ability to stimulate HCEn cells to produce interferon-β (Figure 1). The panel included AGE, high mobility group box 1 (HMGB1), CpG oligonucleotide, and serum amyloid-A (SAA). Our results showed that AGE, HMGB1, and SAA did not significantly stimulate interferon-β expression. However, the CpG oligonucleotide significantly stimulated the expression of interferon-β.

**Figure 2:**
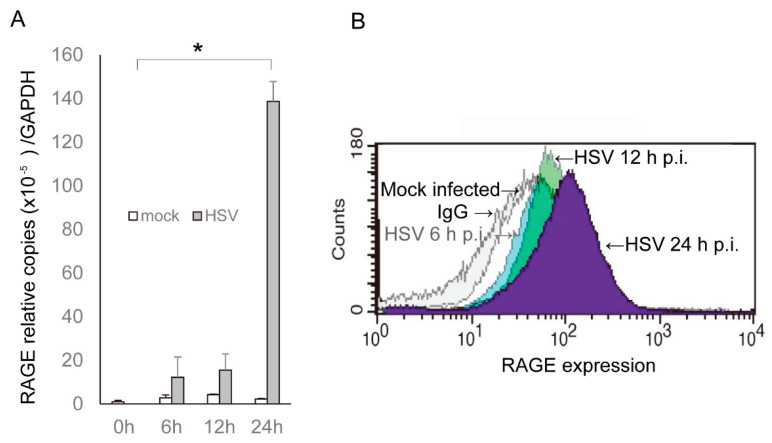
Induction of RAGE expression after HSV-1 infection. HCEn cells were infected with HSV-1 at multiplicity of infection (MOI) of 1 and evaluated for the expression of RAGE. (**A**) The induction of the mRNA of RAGE determined by real-time RT-PCR is significantly increased and peaked at 24 h post infection. *: *p* < 0.00001. *n* = 4. (**B**) Cell surface expression of RAGE after HSV-1 infection was assessed by FACS analysis. RAGE expression is increased after infection and peaked at 24 h. Mean signal intensity of RAGE was 26 ± 1 (mock), 55 ± 3 (6 h, *p* = 0.001), 117 ± 6 (12 h, *p* = 0.000), and 149 ± 7 (24 h, *p* = 0.000). *n* = 374/group.

HSV-1 is the most common pathogen causing corneal endothelial inflammation [17], and the HSV-1 genome contains CpG DNA sites [18]. Therefore, we hypothesized that corneal endothelial cells use RAGE to recognize HSV-1 infections and initiate antiviral interferon responses. We first examined whether HSV-1 infection can transcriptionally induce the expression of the RAGE. To accomplish this, HCEn cells were infected with HSV-1, and the induction of the mRNA of RAGE was determined by real-time RT-PCR (Figure 2A). The results showed that HSV-1 infection stimulated the induction of RAGE at 6 h post-infection (PI), and a 60-fold increase in the induction was observed at 24 h PI, compared to mock infections. 

To determine whether a surface expression of RAGE occurred in HCEn cells, the cell surface was assessed by FACS analysis (Figure 2B). Consistent with the RAGE mRNA induction, HSV infection induced the surface expression of RAGE, which peaked at 24 h PI.

**Figure 3:**
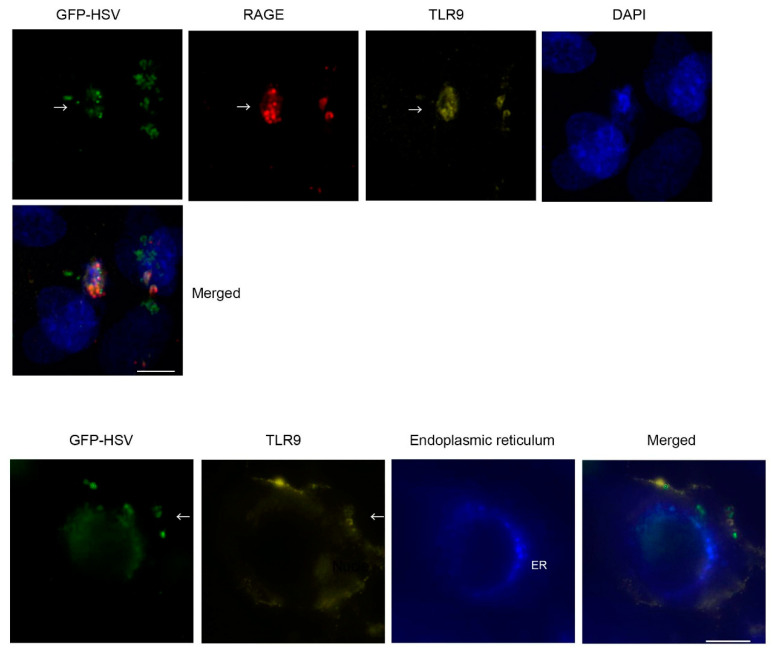
Cellular localization of TLR9, RAGE, and HSV-1. HCEn cells were infected with GFP-HSV (at MOI 50, arrow (green)) and stained for cell surface RAGE (arrow (red): PE-labeled) and TLR9 (Alexa647-labeled, arrow (yellow)) without permeabilization. HSV-1 is colocalized with cell surface RAGE, which partly overlapped with the accumulated TLR9 expression (upper panel). Lower panel: GFP-HSV (arrow (green)) was associated with TLR9 (arrow (yellow)) near cell surface. TLR9 expression was transitioned to endoplasmic reticulum (ER, blue) surrounding nucleus (lower panel). Nuclear transition of GFP-HSV is also observed at 3 h PI. Bar indicates 10 µm.

The CpG DNA sites are mainly recognized by TLR9, and HSV is well known to be recognized by TLR9 [19]. Because this suggested roles for RAGE after HSV infection or CpG stimulation, we hypothesized that the RAGE interacts with TLR9. After HSV-1 infection, HSV-1 is endocytosed or fused with the plasma membrane of the infected HCEn cells. Immunohistochemical analyses showed that the GFP-expressing HSV-1 colocalized with the punctate expression of the cell surface RAGE at 1 h PI. GFP-expressing HSV-1 was also colocalized with TLR9 near the cell surface at 3 h PI, which partly overlapped with RAGE expression. The expression of TLR9, which was associated with GFP-HSV-1, was transitioned to the endoplasmic reticulum structure (Figure 3).

**Figure 4:**
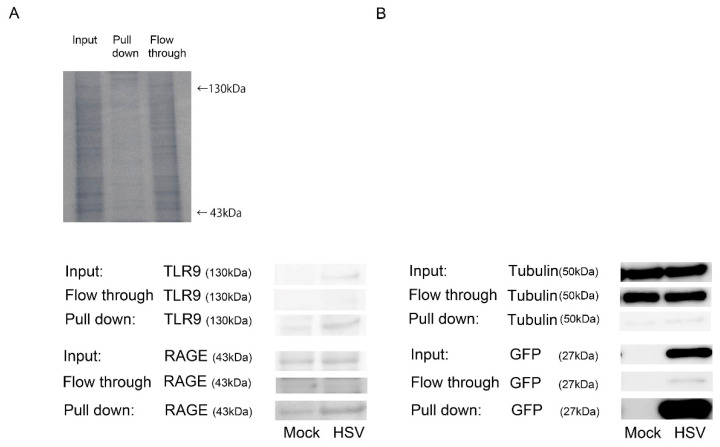
Association of TLR9, RAGE, and HSV-1. (**A**) Association of HSV-1 and RAGE by a GFP-pull-down assay. HCEn cells were infected with HSV-1 at MOI of 50, and the cell lysates as input were pulled down by anti-GFP antibody. The proteins associated with GFP-HSV-1 were detected by SDS PAGE. SDS PAGE, gel stained by Coomassie Blue, is shown as the input, pull down, and flow through fraction (upper panel). To observe RAGE and TLR9, Western blot for each fraction was conducted for RAGE and TLR9 (lower panel). (**B**) Tubulin and GFP in each fraction are shown by Western blot.

To confirm whether HSV-1 is associated with RAGE, HCEn cells were infected with GFP-expressing HSV-1, and the cell lysates were assayed for the GFP pull-down assay (Figure 4). After HSV-1 infection, GFP pulled down RAGE, confirming that HSV-1 is associated with RAGE. GFP was also associated with TLR9.

To determine the role played by RAGE in HSV-1 infections, we examined whether the RAGE could affect viral replication in cultured HCEn cells. HCEn cells were infected with HSV-1 at MOI 1 and were examined for viral titer by RAGE blockade. An inhibition by an anti-RAGE antibody did not significantly affect the viral replication, e.g., HSV-1 titer with control IgG was 4.4 × 10^7^ plaque forming units (PFUs), and that for the anti-RAGE antibody was 4.3 × 10^7^ PFU (NS; N = 4).

**Figure 5:**
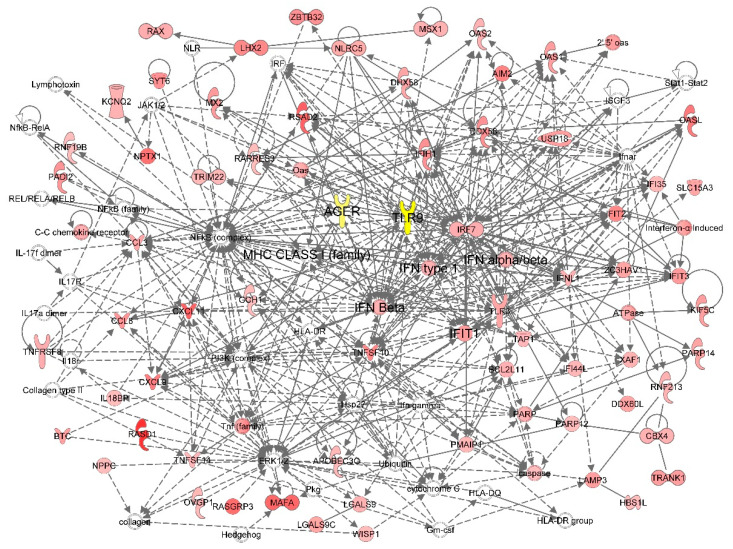
Network analysis of *RAGE* (*AGER*) and HSV-1 infection-induced transcriptional responses of HCEn cells. Functional analysis was conducted to determine the associations of the HSV-1 infection-induced genes with biological functions. A set of 430 infection-induced genes (fold induction > 4) were analyzed for network generation of biological functions. The 3 highest significant networks (*p* < 1 × 10^−32^) are summarized as merged networks. *RAGE* (*AGER*) and *TLR9* were significantly associated with type I interferon responses.

Next, we analyzed the global transcriptional responses of HCEn cells after HSV-1 infection. To accomplish this, we conducted a network analysis, using the highly induced molecules (more than a 4-fold increase) and pattern recognition receptors, TLR9 and RAGE (Figure 5). 

The top canonical pathway was interferon signaling (*p* = 1.1 × 10^−11^), followed by the role of pattern recognition receptors in the recognition of viruses (*p* = 5.8 × 10^−8^). Top upstream regulators were *type 1 interferon*, *interferon lambda-1*, *interferon regulatory factor 7* (*IRF7*), and *STAT1*. Thus, the transcriptional network was highly enriched with a type 1 interferon response and pattern recognition receptors, including *RAGE* and *TLR9*. 

The resultant networks indicated direct associations with *RAGE* and *TLR9* (Figure 5), and their significant contribution to type I interferon responses in recognition of the virus.

**Figure 6:**
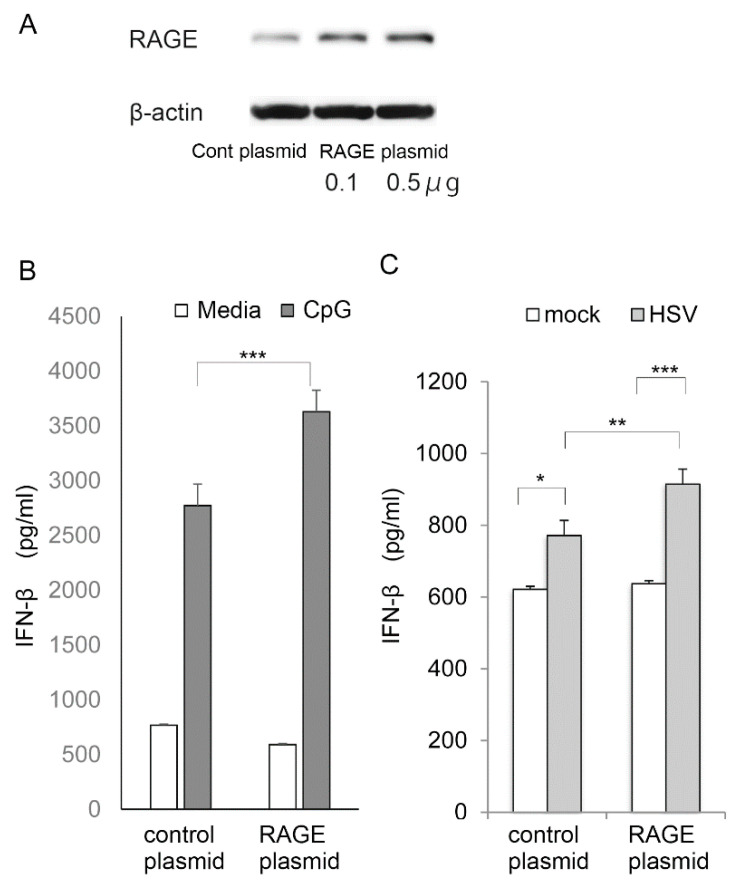
Induction of interferon-β by HCEn cells overexpressing RAGE. HCEn cells were transfected with RAGE-expressing plasmid or control plasmid. Transfection of RAGE c-DNA results in 35kDa, 43kDa, and 70kDa bands by Western blot, reflecting post translational processing [20]. Overexpressed RAGE was shown for 70 kDa band (**A**). RAGE-transfected HCEn cells were assessed for interferon-β secretion 24 h after CpG exposure or HSV-1 infection. Forced induction of RAGE increased the expression of interferon-β by CpG (50 μM) (**B**), and HSV infection at multiplicity of infection (MOI) 1 (**C**). *: *p* = 0.01, **: *p* = 0.001, ***: *p* <0.001; *n* = 6.

We then tested whether RAGE plays a role in the induction of interferon-β. For this, HCEn cells were transfected with RAGE plasmid to overexpress RAGE [20]. The overexpression of RAGE was confirmed by Western blot (Figure 6A). RAGE-transfected HCEn cells were stimulated with CpG DNA or HSV-1 (Figure 6B,C), and the supernatant was assayed for interferon-β secretion. The results showed that the induction of RAGE significantly increased the production of interferon-β by CpG DNA (Figure 6B). 

Next, RAGE-transfected HCEn cells were infected with HSV-1, and the supernatant was examined for interferon-β production (Figure 6C). Again, the RAGE-transfected HCEn cells increased the induction of interferon-β significantly.

**Figure 7:**
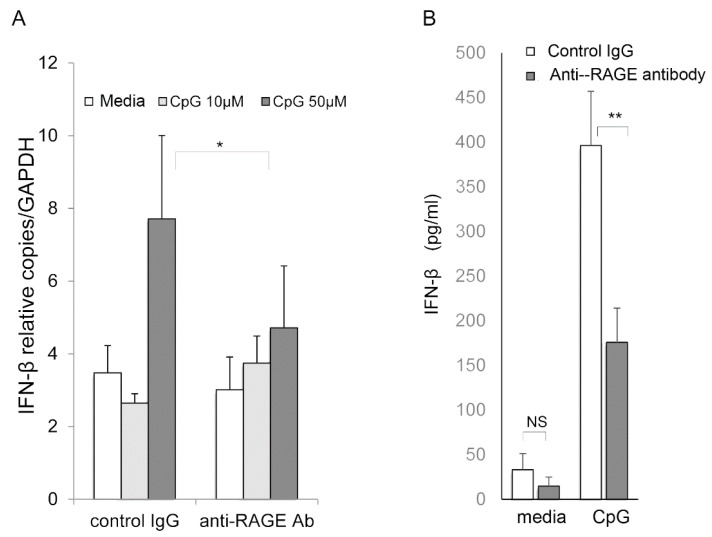
Role of RAGE in interferon-β production by CpG oligonucleotide stimulation of corneal endothelial cells. (**A**) HCEn cells were stimulated by CpG oligonucleotides and were assessed for interferon-β mRNA induction at 12 h by real-time RT-PCR. HCEn cells stimulated by CpG oligonucleotides significantly induced interferon-β. Inhibition by anti-RAGE antibody significantly reduced the induction of interferon-β by CpG oligonucleotide. (**B**) HCEn cells were stimulated by CpG and were assessed for interferon-β production at 24 h by ELISA. The interferon-β production by CpG oligonucleotides (50 μM) was significantly reduced by anti-RAGE antibody. *n* = 6, *: *p* = 0.01, **: *p* < 0.001. Ns: not significant.

We then evaluated the contribution of RAGE to interferon-β induction by CpG DNA. HCEn cells were stimulated with CpG DNA and were examined for the induction of interferon-β. The RAGE blockade significantly reduced the expression of the mRNA of interferon-β (Figure 7A) and the protein (Figure 7B) induced by CpG DNA.

**Figure 8:**
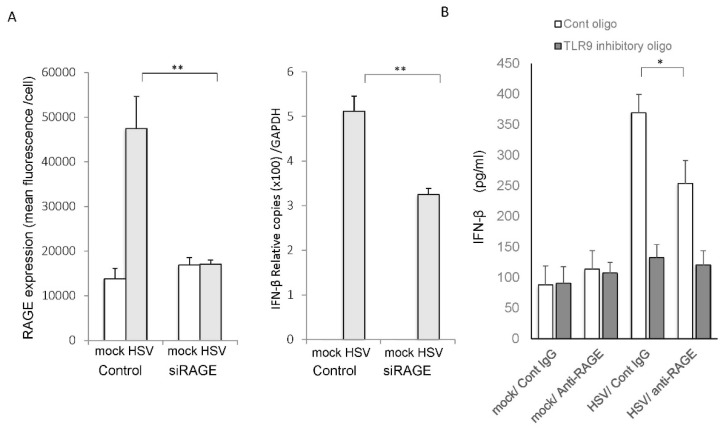
Role of RAGE and TLR9 in the interferon-β production by HSV-1 infection of corneal endothelial cells. (**A**) HCEn cells were transfected by siRNA of RAGE or control siRNA, and they were assessed for cell surface expression of RAGE with or without HSV-1 infection (multiplicity of infection (MOI) 1. Plated cells were stained for the surface expression of RAGE, and 1300 cells in high powered field per group were assessed for total fluorescence intensity. Si RNA transfection abolished HSV-1-induced-RAGE expression. HCEn cells were infected by HSV-1 infection at MOI 1 and were assessed for the expression of the mRNA of interferon-β at 12 h by real-time RT-PCR. The HSV-1 infection induced-interferon-β mRNA were significantly impaired by the RAGE blockade using siRAGE. (**B**) HCEn cells were infected by HSV-1 and were assessed for interferon-β production at 24 h by ELISA. HCEn cells stimulated with HSV-1 infection at MOI 1 induced interferon-β which was significantly reduced by anti-RAGE antibody and abolished by TLR9 inhibitory oligonucleotide (2 µM). *: *p* < 0.01; **: *p* <0.001; *n* = 6.

Next, we evaluated the contribution of RAGE to the induction of interferon-β after HSV-1 infection. HCEn cells were inhibited for the expression of RAGE and were assessed for interferon-β induction after HSV-1 infection. HCEn cells were transfected with RAGE siRNA and were assessed for cell surface expression of RAGE (Figure 8A). The transfection of siRNA abolished HSV-induced upregulation of the RAGE expression. 

When the RAGE siRNA-transfected HCEn cells were infected with HSV-1, the induction of the mRNA of interferon-β was significantly reduced (Figure 8A). 

Next, we examined how TLR9 is involved in the RAGE-mediated interferon-β expression. The level of HSV-1 infection-induced interferon-β was significantly reduced after a RAGE blockade (Figure 8B). Inhibition of TLR9 abolished the HSV infection-induced interferon-β secretion from HCEn cells. Collectively, RAGE is involved in signaling by HSV-1 infection of interferon-β induction, which requires TLR9.

## 3. Discussion

Apart from aging, viral endotheliitis is an important risk factor for corneal endothelial cell loss, which then leads to bullous keratopathy [21]. Both aging and viral infections, including HSV-1 and cytomegalovirus, are strongly associated with type I interferon responses manifested as inflammation. Alternatively, the natural course of senescence can cause cell damage as sterile inflammation. 

We examined how RAGE contributed to endothelial cell defense and showed that an important aspect of RAGE was as a sensor of HSV-1 infection and the induction of type 1 interferon responses.

Viral corneal endotheliitis is typically caused by infections by HSV-1, CMV, or varicella-zoster virus [10,11,12,22]. The viral infections are initially recognized by pattern recognition receptors as part of the innate immune system, including TLR-like receptors, NOD-like receptors, and scavenger receptors [23]. The signaling by these receptors typically results in type I interferon responses or NF-κB activation. When HSV-1 was assessed for recognition by pattern recognition receptors of corneal endothelial cells, TLR9 played a pivotal role in inducing the interferon response [16]. However, TLR9 is located endosomally and requires signaling input from the cell surface receptors. We showed that the cell surface RAGE contributed to HSV recognition by corneal endothelial cells and route signaling to TLR9. 

AGE can be formed by the collagen or elastin of the extracellular matrix in the skin of elderly individuals [24]. This can also occur in the Descemet’s membrane of diseased cornea or FECD [8], and the altered extracellular matrix environment can signal back to the endothelial cells through RAGE [25]. 

In the cornea of elderly FECD patients, AGE is known to accumulate abundantly in the stressed or thickened anterior portion of basement membrane (Descemet’s membrane) of corneal endothelial cells accompanied by endothelial cell loss [8]. This suggests that accumulated AGEs can cause significant cellular stress that contributes to endothelial cell loss. Accumulated AGEs need to be removed by scavenger receptors, including RAGE. However, eyes with FECD are impaired in the expression of RAGE. In normal elderly individuals, AGE is also observed in the cornea; however, its deposition in the corneal endothelium is limited to a scattered manner. 

RAGE shares several ligands with TLRs, including HMGB1, S100, and LPS, and its signaling cascade involve MyD88, which is the key intracellular adaptor of TLR9 signaling (Figure 5) [26,27]. TLR9 typically recognizes CpG DNA and HMGB1, which are also ligands for RAGE. In addition, RAGE crosstalks or cooperates with TLR9 (Figure 5). RAGE forms complexes with TLR9 to recognize pathogenic DNA leading to autoimmune responses [28]. Our transcriptional analysis indicated that TLR9 was associated with a number of type I interferon-associated molecules, including *IFNB1*, *IFIH1*, *IFN beta*, *IRF1*, *IRF4*, *IRF7*, *IFIT2*, *IFIT3*, *IFI35*, *IFITM1*, and *ISG20* (Figure 5). 

Infections by HSV-1 also require cell surface receptors; heparan sulfate on the cell membrane initially binds glycoproteins B and C of the HSV-1 envelope and this plays a role in the initial attachment [29]. Interestingly, heparan sulfate has been proposed as a co-receptor of RAGE binding to HMGB1 [30]. 

We observed an increase in the surface expression of RAGE in HCEn cells after HSV-1 infection, and this may allow their higher responsiveness. TLR9 is indispensable for sensing CpG DNA or HSV-1 infections even when RAGE is functioning. This is consistent with the findings that cell surface RAGE is located upstream of endosomal TLR9, and RAGE appears to help route the viral stimulus to endosomal TLR9. This can then decrease the threshold of cellular activation and allow responses to viral infections [31]. 

Another property of RAGE is its ability to be directly associated with released DNA or RNA from damaged cells on the cell surface [32]. This is in marked contrast to TLR9, which is located in the endosome and appears to not be readily activated. 

The RAGE in corneal endothelial cells plays multiple roles, including that of scavengers and antiviral receptors. For example, IL-6 is a typical scavenger receptor-induced cytokine. Scavenger receptors type A, including *COLEC12*, *MARCO*, *MSR1*, *SCARA3*, and *SCARA5*, induce IL-6 (Figure 5). We observed that AGE stimulation of corneal endothelial cells induced IL-6 (Figure 5). However, AGE did not stimulate interferon-β (Figure 1). The RAGE-induced-interferon-β appears restricted to TLR9-stimulating input. The presumed dual roles of RAGE may affect the endothelial physiology. In FECD patients, the expression of RAGE in the corneal endothelial cells is reduced [8]. This impairs its scavenger function as well as the antiviral responses when they are affected by a primary or reactivated HSV-1 infection. 

The type 1 interferon response is an efficient antiviral response; however, chronic and intermittent responses may become damaging for corneal endothelial cells. One way for this is through collateral damage by type 1 interferon-activated cytotoxic effector CD8^+^ T cells [33]. Another way is the promotion of cell senescence due to a shortening of the telomere length by prolonged interferon stimulation [34].

To treat viral infection-related endothelial inflammation, topical antiviral drugs are suggested to prevent progressive endothelial cell loss. Failure to control viral activity can lead to the need for corneal endothelial cell transplantation. Currently, it remains unclear whether strategies to remove AGE or prevent RAGE signaling may have clinically beneficial effects. This requires future clinical trials.

Collectively, RAGE not only functions as a recognizer of age-related oxidized products or AGEs, but it also contributes to antiviral responses as the innate immune arm. 

There are some limitations in this study. We reported that the cytokine responses of our HCEn cells were very similar to those of primary cultured corneal endothelial cells [33,35,36], and confirmed RAGE expression or strong induction of type 1 interferon by primary endothelial cells after HSV-1 infection or TLR9 stimulation (data not shown). We are aware that HCEn cells may not exactly mirror the physiological functions of primary endothelial cells. However, a subculturing of primary corneal endothelial cells is difficult, as only low-passaged cells replicate. This significantly affects the reproducibility of HSV-1 infections after plasmid transfection or manipulation. 

The function of RAGE is presumably context-dependent, and a complete picture of the contributions of RAGE to diseases appears more complex than believed. Importantly, RAGE recognized CpG DNA in conjunction with TLR9 and induced antiviral interferon responses in corneal endothelial cells. This information may help develop therapy to treat viral corneal endotheliitis or senescence-related endothelial cell dysfunction.

## 4. Materials and Methods

### 4.1. Cells

The immortalized human corneal endothelial cell line (HCEn), established by transduction with hTERT and the large T gene, were propagated to confluence in Dulbecco’s modified Eagle’s medium (DMEM; Gibco, Grand Island, NY, uSA) supplemented with 10% fetal bovine serum (FBS) (Gibco, ThermoFisher Scientific, Waltham, MA, USA), [15,16,35]. HCEn cells were seeded in 96-well plate (2 × 10^4^ cells/well) and stimulated with panels of RAGE ligands, including unmethylated CpG oligonucleotide (ODN2216, class A, InVivoGen, San Diego, CA, USA) [37,38,39,40,41], AGEs (BioVision, Milpitas, CA, USA), recombinant high mobility group box-1 (HMGB1; R&D, Minneapolis, MN, USA), and serum amyloid A (SAA) in 10% FBS DMEM. The activity of ODN2216 (InVivoGen) on TLR9 of corneal endothelial cells was previously confirmed using control ODN2243 (GpC oligonucleotide, ODN2243) [16], which did not induce TLR9 activity [16,39]. The absence of LPS contamination in the ODNs was assayed, using TLR4 reporter cells.

### 4.2. Virus

HSV-1 viral stocks were prepared from confluent monolayers of Vero cells infected with the KOS strain or GFP-KOS strain (generously gifted from Kozaburo Hayashi and David Tscharke) of HSV-1 [42]. Purified viral stocks were prepared using a sucrose density gradient as described in detail [42]. The samples were aliquoted and stored at −80 °C until use. 

### 4.3. ELISA

The level of interferon-β in the supernatant was measured with an ELISA kit (ANTIGENIX America, Huntington Station, NY, USA).

### 4.4. Real-Time RT-PCR

Total RNA was isolated from the HSV-1-infected and non-infected HCEn cells and reverse transcribed, using the QuantiTect Reverse Transcription Kit (Qiagen, Hilden, Germany). The cDNAs were amplified and quantified by the LightCycler (Roche, Mannheim, Germany), using the QuantiTect SYBR Green PCR kit. The sequences of the real-time PCR primer pairs were as follows:

RAGE:

forward 5′-TGGAACCGTAACCCTGACCT-3′

reverse 5′-CGATGATGCTGATGCTGACA-3′

 

interferon-β:

forward 5′-CATTACCTGAAGGCCAAGGA-3′

reverse 5′-CAATTGTCCAGTCCCAGAGG-3′

 

GAPDH:

forward 5′-AGCCACATCGCTCAGACAC-3′

reverse 5′-GCCCAATACGACCAAATCC-3′

### 4.5. Fluorescence-Activated Cell Sorting (FACS) Analyses

HCEn cells were seeded in 3.5 cm dishes with a temperature-responsive surface (5 × 10^5^ cells/dish, UpCell, CellSeed, Tokyo, Japan) and infected with HSV-1. Cells were harvested without trypsinization and stained with mouse anti-RAGE antibody (ab89911, Abcam, Cambridge, UK) and made visible by DyLight 488-conjugated secondary antibody. FACS analysis was conducted using FACS Calibur (Becton Dickinson, Franklin Lakes, NJ, USA).

### 4.6. Immunohistochemistry

HSV-1-infected HCEn cells were seeded onto 8 chamber glass slides (5 × 10^5^ cells/chamber, Corning, NY, USA). The cells were fixed in 1% paraformaldehyde and were stained with a mouse anti-RAGE antibody (ab89911, Abcam, Cambridge, UK) or rabbit anti-human TLR9 (bs-2717R, Bioss, Woburn, MA, USA) and made visible by Alexa 647 (Thermo Fisher Scientific, Waltham, MA, USA) or PE-conjugated secondary antibody (Santa Cruz Biotechnology, Dallas, TX, USA), respectively. The endoplasmic reticulum was stained using a live cell endoplasmic reticulum staining kit (Blue fluorescence, AAT Bioquest, Sunnyvale, CA, USA). The cells were photographed and analyzed using a fluorescent microscope with hybrid cell count and sectioning software (BZ-X810, Keyence, Osaka, Japan).

### 4.7. GFP Pull-Down Assay

HCEn cells were infected with GFP-HSV-1 at multiplicity of infection (MOI) of 50 and lysed for immunoprecipitation, using lysis buffer (10 mM Tris/Cl pH 7.5, 150 mM NaCl, 0.5 mM EDTA, 0.5% Nonidet P40 substitute, and 0.09% sodium azide). The lysates were incubated with agarose beads covalently coupled with alpaca anti-GFP antibody fragments (GFP-Trap_A, chromotek, Planegg-Martinsried, Germany) according to the manufacture’s instruction. The beads were washed with wash buffer (10 mM Tris/Cl pH 7.5, 150 mM NaCl, 0.5 mM EDTA, 0.05% Nonidet P40 substitute, and 0.018% sodium azide) three times. Proteins bound to GFP and anti-GFP antibody were separated by SDS polyacrylamide gel electrophoresis (PAGE) and detected by immunoblot. For the detection of RAGE, TLR9, and GFP, mouse anti-RAGE antibody (clone A17158D, Biolegend), rabbit anti-TLR9 antibody (#5845, Cell Signaling), and mouse anti-GFP antibody (clone 1GFP63, Biolegend, San Diego, CA, USA) were used.

### 4.8. Transfection of RAGE Expression Plasmid and Blockade of RAGE 

HCEn cells were transfected with a RAGE (NM_001136)-expressing plasmid with a CMV promoter (SC122583, pCMV6 backbone, OriGene, Rockville, MD, USA), using gene porter 3000 [20]. The extracted proteins were confirmed by Western blot, using anti-RAGE antibody and HRP-conjugated secondary antibody (Cell Signaling, Danvers, MA, USA). A mouse anti-RAGE antibody (ab89911, clone MM0520-8D11, Abcam) or siRNA transfection was used to inhibit RAGE. The neutralization function of the mouse anti-RAGE antibody (ab89911) was confirmed, using functional ELISA. When Biotinylated-AGE-BSA (0.5 μg/mL) was tested, the anti-RAGE antibody (10 μg/mL) blocked 92% of the binding.

For the siRNA transfection, HCEn cells were transfected with RAGE siRNA (Silencer Select Pre-designed siRNA, s1166 (4392420), ambion, Thermo Fisher Scientific) with RNAifect (Qiagen, Hilden, Germany) according to the manufacturer’s instructions. For inhibition of TLR-9, TLR-9 inhibitory oligonucleotide (5′-tcctggcggggaagt-3′) was used (2 µM, Alexis, San Diego, CA, USA). Neutral oligonucleotide (5′-tgctcctggaggggttgt-3′) was used as the control.

### 4.9. Network Analysis of Corneal Endothelial Cell Transcriptome after HSV-1 Infection

HCEn cells were infected with HSV-1 (KOS strain) at MOI 1 and the mRNAs were extracted 12 h post-infection [15]. The transcriptome data obtained using whole human genome microarray (Agilent Technologies, Santa Clara, CA, USA) were analyzed [15]. To identify the canonical pathways and transcriptional networks that were most significant to the dataset, the data were analyzed by the Ingenuity Pathway Analysis (IPA; 2018 winter version, QIAGEN Inc. accessed March 2019)

### 4.10. Statistical Analyses

Data are presented as the means ± standard error of the means (SEMs). The significance of the differences was determined by *t* tests or ANOVA and post hoc tests.

## Data Availability

The data that supports the findings of this study are available within the article.

## Abbreviations

HSV-1	herpes simplex virus type 1
PAMP	pathogen-associated molecular patterns
RAGE	receptor for advanced glycosylation end products
AGE	advanced glycation end products
HCEn	human corneal endothelial cell
HMGB1	high mobility group box 1
TLR9	toll-like receptor 9
SAA	serum amyloid-A
PI	post-infection
